# Direct training high-performance spiking neural networks for object recognition and detection

**DOI:** 10.3389/fnins.2023.1229951

**Published:** 2023-08-08

**Authors:** Hong Zhang, Yang Li, Bin He, Xiongfei Fan, Yue Wang, Yu Zhang

**Affiliations:** ^1^State Key Laboratory of Industrial Control Technology, College of Control Science and Engineering, Zhejiang University, Hangzhou, China; ^2^Key Laboratory of Collaborative Sensing and Autonomous Unmanned Systems of Zhejiang Province, Hangzhou, China

**Keywords:** spiking neural networks, gate residual learning, attention spike decoder, spiking RetinaNet, object recognition, object detection

## Abstract

**Introduction:**

The spiking neural network (SNN) is a bionic model that is energy-efficient when implemented on neuromorphic hardwares. The non-differentiability of the spiking signals and the complicated neural dynamics make direct training of high-performance SNNs a great challenge. There are numerous crucial issues to explore for the deployment of direct training SNNs, such as gradient vanishing and explosion, spiking signal decoding, and applications in upstream tasks.

**Methods:**

To address gradient vanishing, we introduce a binary selection gate into the basic residual block and propose spiking gate (SG) ResNet to implement residual learning in SNNs. We propose two appropriate representations of the gate signal and verify that SG ResNet can overcome gradient vanishing or explosion by analyzing the gradient backpropagation. For the spiking signal decoding, a better decoding scheme than rate coding is achieved by our attention spike decoder (ASD), which dynamically assigns weights to spiking signals along the temporal, channel, and spatial dimensions.

**Results and discussion:**

The SG ResNet and ASD modules are evaluated on multiple object recognition datasets, including the static ImageNet, CIFAR-100, CIFAR-10, and neuromorphic DVS-CIFAR10 datasets. Superior accuracy is demonstrated with a tiny simulation time step of four, specifically 94.52% top-1 accuracy on CIFAR-10 and 75.64% top-1 accuracy on CIFAR-100. Spiking RetinaNet is proposed using SG ResNet as the backbone and ASD module for information decoding as the first direct-training hybrid SNN-ANN detector for RGB images. Spiking RetinaNet with a SG ResNet34 backbone achieves an mAP of 0.296 on the object detection dataset MSCOCO.

## 1. Introduction

In recent years, significant progress has been made in deep learning research, which has become a primary tool for various computer vision tasks, such as object recognition, object detection, and semantic segmentation. Key technologies such as ResNet (He et al., 2016) and batch normalization (Ioffe and Szegedy, 2015) have enabled the construction of deep neural networks with numerous parameters and deep model structures, achieving high accuracy in the aforementioned tasks. However, the growing network complexity and data quantity make it increasingly expensive to train and deploy deep neural networks. Therefore, it is necessary to explore network models and computational paradigms that are more efficient than current artificial neural networks (ANNs). One of the main research directions is the spiking neural network (SNN), a bionic neuron model inspired by biological neuron models based on spiking signals (Gerstner and Kistler, 2002; Cheng et al., 2023; Yi et al., 2023). Researchers have paid considerable attention to SNN because of its high-energy efficiency on neuromorphic hardwares (Merolla et al., 2014; Davies et al., 2018).

Due to the non-differentiability of the spiking signals, training high-performance SNNs is challenging. First, researchers utilized the spike-timing-dependent plasticity (STDP) (Song et al., 2000) rule to conduct the unsupervised training of SNNs. STDP is a biology-inspired process that adjusts the synaptic weights based on the relative timing of the presynaptic and postsynaptic neurons' action potentials. However, STDP cannot accomplish supervised learning for large-scale networks, which limits its practical application. Currently, there are two mainstream approaches to obtain deep SNN models: ANN-to-SNN conversion and direct-training. The ANN-to-SNN conversion method consists of two steps. First, an ANN model corresponding to the target SNN model is trained. Then, the connection between the firing rates of the SNN and the activation values of the ANN are used to establish a conversion formula to help convert the weight of the ANN model to that of the SNN. The accuracy of this conversion is largely determined by the simulation time steps of SNNs. The simulation time step is usually in hundreds or thousands to obtain an SNN with competitive performance, which results in the unacceptably high latency. The second method, direct-training, approximates the non-differentiable heaviside step function with a surrogate gradient and trains the SNNs directly through backpropagation. Researchers usually adopt the backpropagation through time (BPTT) framework, which is derived from RNN. Unlike ANN-to-SNN conversion, the direct-training method requires only a tiny time step. The network thus obtained has very low latency, making it superior in real-time scenarios. However, because of the complicated neural dynamics of SNNs and the non-differentiability of spiking signals, direct-training of SNNs requires further exploration on several crucial issues to achieve acceptable results on large-scale datasets, such as ImageNet (Deng et al., 2009) and MSCOCO (Lin et al., 2014).

The first issue is the gradient vanishing or explosion problem, which restricts SNNs to shallow architectures. To solve this problem, a natural idea is to introduce residual learning from ANNs into the SNNs. Spiking ResNet (Lee et al., 2020) replaces the ReLU activation function in the residual block with spiking neurons such as the integrate-and-fire (IF) and leaky-integrate-and-fire (LIF) (Gerstner and Kistler, 2002). However, it has been found that such a spiking residual block of spiking ResNet cannot achieve identity mapping because of the complex dynamics of spiking neurons. On this basis, SEW ResNet (Fang et al., 2021a) has used an element-wise function to modify the residual block and has successfully achieved identity mapping. However, the ADD function used in SEW ResNet introduces non-spiking signals, which no longer conforms the properties of SNNs and preventes SNNs from being deployed on neuromorphic hardware. Therefore, effective residual learning in SNNs remains a problem worth exploring. We believe that the shortcut connection with addition in the residual block enables the analog tensor in different levels to achieve lossless information fusion, which is the reason for the high performance of the ADD-based SEW ResNet. However, the spiking signal is binary, and its addition operation cannot be deployed. This motivates us to explore a better residual block structure to accomplish information fusion with only full-spike operations.

Another problem is the decoding of spike trains, which determines the high-dimensional image features in object recognition. There are two schemes: temporal and rate coding. The former directly adopts spike times as the information carrier, which is efficient in large time-step systems. However, direct-training SNNs have tiny time steps, leading to low accuracy of temporal coding. The latter method uses firing rates as the information carrier. Many direct training methods (Fang et al., 2021a; Zheng et al., 2021) have adopted it due to its high performance. However, Wu et al. (2021) found that rate coding produced a less smooth learning curve, reducing the final accuracy. Meanwhile, from the perspective of neuroscience, rate coding is unreasonable because it treats activation at each time step as equally important. In fact, spikes at different times and in different spaces may have different effects on the results, depending on the salient region (Itti et al., 1998; Yao et al., 2021).

The inability to handle complex computer vision tasks well is another problem. Most existing approaches are limited to classification. Object detection, a fundamental task in vision, has widespread applications in many real-time scenarios. However, there are only a few direct-training spiking object detectors. Cordone et al. (2022) trained an SSD detector using spiking VGG, MobileNet, and DenseNet as the backbones. Kugele et al. (2021) constructed a similar detector using a spiking DenseNet. They both performed detection on event data. Neither case performed well in the large-scale MSCOCO datasets, indicating that their methods were not applicable to most existing vision systems. In addition, the gradient degradation problem was not addressed such that the deeper DenseNet achieved a worse accuracy in Cordone et al. (2022).

To address the gradient vanishing or explosion problem, we implemented the identity mapping of the residual block under the constraints of spiking signals by proposing spiking gate (SG) ResNet. The inspiration mainly comes from GRU (Cho et al., 2014) and Highway Network (Srivastava et al., 2015). These works have shown that the gate mechanism can dynamically control the flow of information in the network and can significantly solve the gradient vanishing problem. In each basic block of SG ResNet, a binary selection gate is introduced to guide the information fusion of the spiking signals. As for the decoding scheme, we propose the attention spike decoder (ASD) to decode the spike output from SG ResNet more effectively. The ASD block is highly generalizable and can be applied to object recognition and detection tasks. The effectiveness of the SG ResNet and ASD block are evaluated on object recognition datasets, including three static image datasets and a neuromorphic dataset. In addition, we propose spiking RetinaNet using SG ResNet as the backbone and the ASD block for information decoding. This is the first direct-training hybrid SNN-ANN detector that can achieve good performance on the MSCOCO dataset.

Our contributions are as follows:

- A spiking gate ResNet with full-spike operations is developed to solve the gradient vanishing in SNNs to make deep SNNs trainable. Furthermore, two appropriate formulations of the binary gate in SG ResNet are provided.
- An attention spike decoder is proposed to apply temporal, channel, and spatial attention to accumulate the information of spiking signals. This is an effective and general decoder for both object recognition and detection.
- Numerous experiments are conducted on both static image and neuromorphic datasets in the object recognition task to verify the effectiveness of the SG ResNet and ASD block.
- Spiking RetinaNet, which is a hybrid neural network, is proposed to combine the SG ResNet backbone with a detection head. The ASD block plays a vital role in spike decoding. We demonstrate that with a proper backbone and decoding, a direct-training SNN can perform well in object detection.

## 2. Related work

There are two main approaches to training and deploying deep SNNs: ANN-to-SNN conversion and direct-training SNNs. Most works of these two approaches restrict the task to object recognition. In this study, a spiking RetinaNet detector is also built. Thus, related works of object recognition will be chiefly overviewed and then we will review the research on object detection with SNNs.

### 2.1. ANN-to-SNN conversion

Rueckauer et al. (2016) provided a theoretical basis for the ANN-to-SNN conversion approach. Theoretically, the firing rates of spiking neurons in SNNs can be estimated by the activation of the ReLU function in ANNs with the corresponding structures. With weight normalization and BN integration, a well-trained ANN can be converted to an SNN with minimal loss of precision (Diehl et al., 2015). Sengupta et al. (2019) proposed SpikeNorm to improve conversion, which was the first to test this approach on deep architectures such as VGG and ResNet. Furthermore, time-based coding (Han and Roy, 2020), SNN calibration (Li et al., 2021), and clamped and quantized training (Yan et al., 2021) have been proposed for optimizing the conversion error. Other methods (Deng and Gu, 2021; Bu et al., 2022; Li and Zeng, 2022) have realized high-performance conversions, which have narrowed the gap between the SNNs and ANNs. However, there are many drawbacks to ANN-to-SNN conversion methods. First, it does not work with neuromorphic data because ANNs cannot be trained on these data. Second, high precision requires SNNs to adopt a very large simulation time step, leading to high latency of the converted SNNs, which is unsuitable for deployment in real scenarios.

### 2.2. Direct-training SNNs

Direct-training methods optimize the parameters of SNNs using direct error backpropagation. One popular approach is to unfold the network over the simulation time by referring to the BPTT framework of the RNN. Because the heaviside step function used in spiking neurons is not differentiable, researchers typically use sigmoid, arc-tangent, and other functions to calculate the surrogate gradient. Such methods (Wu et al., 2018; Neftci et al., 2019; Lee et al., 2020) usually obtain low latency and can train SNNs with small simulation time steps. Recently, in addition to designing powerful spiking neurons (Fang et al., 2021b; Li et al., 2022; Yao et al., 2022), researchers have primarily improved the accuracy of direct-training SNNs from network structure and training techniques (Guo et al., 2023). We will elaborate on them.

Similar to ANN, deep plain SNNs still suffer from gradient vanishing or explosion problems. Thus, SNN structure design based on residual learning has become mainstream. However, indiscriminately imitating the ResNet (Lee et al., 2020) cannot solve this problem because of the properties of spiking neurons. Fang et al. (2021a) systematically analyzed the cause of gradient vanishing from the perspective of identity mapping in SEW ResNet and tried to solve this problem using element-wise functions. However, their approach breaks the rule that SNNs use only spiking signals. In addition, multi-level firing (Feng et al., 2022), membrane shortcut (Hu et al., 2021), and threshold-dependent batch normalization (Zheng et al., 2021) technologies have been successively utilized to construct deeper SNNs. Unlike the manually designed networks above, AutoSNN (Na et al., 2022) and SNASNet (Kim et al., 2022) use the neural architecture search approach for SNN structure design.

At the training technique level, some researchers aim to improve the surrogate gradient-based backpropagation process. These improvement routes include membrane or spike regularization (Guo et al., 2022a,c), spike knowledge distillation (Xu et al., 2023a,b), and designing better surrogate gradients (Che et al., 2022; Guo et al., 2022b). In addition to the surrogate gradient-based backpropagation, there are also some effective direct-training methods designed specifically for SNNs, including STDP-based learning (Saunders et al., 2018; Tavanaei and Maida, 2019; Hao et al., 2020), tandem learning (Wu et al., 2021), and differentiations on spike times (Mostafa, 2017; Wunderlich and Pehle, 2021; Zhou et al., 2021), spike representation (Meng et al., 2022), and equilibrium state (Xiao et al., 2021).

### 2.3. Object detection with SNNs

Similar to object recognition, spiking detectors are generated by ANN-to-SNN conversion and direct-training methods. Spiking YOLO (Kim et al., 2020) is the first spiking detector converted from an ANN. Channel-wise data-based normalization is used to optimize the conversion process. Miquel et al. (2021) proposed the analog-to-spiking conversion method, which allows a more complex network structure like RetinaNet. Chakraborty et al. (2021) combined the unsupervised spike time dependent plasticity method and hybrid training method spike time dependent backpropagation to deploy a spiking hybrid detector. All the above studies have adopted conversion from ANN to SNN. Therefore, their networks have large time steps and are inefficient. Kugele et al. (2021) and Cordone et al. (2022) presented direct-training spiking detectors by combining some spiking backbones with an SSD detection head. Such detectors have very low latency and are well-suited for real-time scenarios. However, they are only applied to event cameras and cannot be used in existing vision systems with RGB camera images. To the best of our knowledge, our spiking RetinaNet is the first to complete object detection on static images dataset such as MSCOCO.

## 3. Spiking architectures and decoding

In this section, we first introduce the spiking neuron models used in this study. Then, the spiking gate residual (SGR) block and SG ResNet is proposed based on residual learning and gate mechanism. We further propose two appropriate representations of the binary selection gate in the SGR block and analyze its gradient propagation. Finally, the attention spike decoder is proposed to decode the spike output from SG ResNet.

### 3.1. Spiking neuron model

Spiking neurons imitate biological neural mechanisms that communicate via spiking signals. The IF and LIF (Gerstner and Kistler, 2002) models are used in this study. They are simplified models with high implementation efficiency while preserving sufficient biological dynamics. The following formula expresses the linear differential equation of LIF neurons:


(1) $$\begin{array}{l} \tau_{\text{m}}\frac{dV_{i}^{l}\left(t\right)}{dt}=-\left[V_{i}^{l}\left(t\right)-V_{rest}\right]+RI_{i}^{l}\left(t\right), \end{array}$$

where $V_{i}^{l}$ is the membrane potential, $I_{i}^{l}$ is the input current, τ_m_ is the time constant, *V*_*rest*_ represents the resting potential, and *R* represents the membrane resistance. Without loss of generality, we treat *R* as unitary in the rest of the study; *i* and *l* denote that this neuron is the *i*_*th*_ one in the *l*_*th*_ layer of the whole network. Compared with LIF, the IF neuron ignores the leaky effect of membrane potential. The following equation describes its dynamics:


(2) $$\begin{array}{l} \frac{dV_{i}^{l}\left(t\right)}{dt}=RI_{i}^{l}\left(t\right). \end{array}$$

In this equation, provided that the membrane potential $V_{i}^{l}$ exceeds the spike fire threshold *V*_*th*_, the neuron fires a spike immediately. Simultaneously, the membrane potential will be reset to *V*_*rest*_. The neuron's output can be then represented by the following equation:


(3) $$\begin{array}{l} s_{i}^{l}\left(t\right)=\text{Θ}\left(V_{i}^{l}\left(t\right)-V_{th}\right) \end{array}$$

where $s_{i}^{l}$ is the output spike of the neuron at time step *t*, and Θ is the heaviside step funcion defined as follows:


(4) $$\begin{array}{l} \text{Θ}(x)=\{\begin{array}{cc} 1, & x\geq 0\\ 0, & x<0 \end{array} \end{array}$$

In practice, it is necessary to discretize the dynamical equations. The discretized LIF and IF dynamics are shown in Eqs 5 and 6 (Fang et al., 2021b), respectively.


(5) $$\begin{array}{l} H_{i}^{l}\left[t\right]=V_{i}^{l}\left[t-1\right]+\frac{1}{\tau_{m}}\left(I_{i}^{l}\left[t\right]-\left(V_{i}^{l}\left[t-1\right]-V_{reset}\right)\right) \end{array}$$

(6) $$\begin{array}{l} H_{i}^{l}\left[t\right]=V_{i}^{l}\left[t-1\right]+I_{i}^{l}\left[t\right] \end{array}$$

where $H_{i}^{l}\left[t\right]$ can be regarded as the hidden membrane potential before trigger time *t*. $V_{i}^{l}\left[t-1\right]$ represents the membrane potential of a neuron at time *t*−1. $I_{i}^{l}\left[t\right]$ is the synaptic current at time *t*, which is determined by the output of the neurons in the preceding layer. $w_{ij}^{l-1}$ denotes the synaptic connection strength between *j*_*th*_ neuron in layer *l*−1 and the *i*_*th*_ neuron in layer *l*, and $b_{i}^{l}$ represents the corresponding bias. Then, $I_{i}^{l}\left[t\right]$ can be expressed by the following formula.


(7) $$\begin{array}{l} I_{i}^{l}\left[t\right]=\underset{j}{\sum }w_{ij}^{l-1}S_{j}^{l-1}\left[t\right]+b_{i}^{l} \end{array}$$

The discrete output equation of the neuron is shown in Eq. 8, where $S_{i}^{l}\left[t\right]$ is the output spiking signal at time *t*. When $S_{i}^{l}\left[t\right]=1$, the neuron fires a spike; otherwise, when ($S_{i}^{l}\left[t\right]=0$), the neuron does not fire any spike.


(8) $$\begin{array}{l} S_{i}^{l}\left[t\right]=\text{Θ}\left(H_{i}^{l}\left[t\right]-V_{th}\right) \end{array}$$

Once the neuron fires a spike, the membrane potential $V_{i}^{l}\left[t\right]$ at time *t* is reset to *V*_*rest*_. Therefore, the discrete representation of $V_{i}^{l}\left[t\right]$ is presented as follows.


(9) $$\begin{array}{l} V_{i}^{l}\left[t\right]=H_{i}^{l}\left[t\right]\left(1-S_{i}^{l}\left[t\right]\right)+V_{rest}S_{i}^{l}\left[t\right]. \end{array}$$

For simplicity, both *V*[0] and *V*_*rest*_ are set to 0. Meanwhile, the derivative of the function Θ is determined by the pre-defined surrogate function. The implementation and GPU acceleration of all neurons are based on the PyTorch and SpikingJelly (Fang et al., 2020) frameworks.

### 3.2. Spiking gate ResNet

**Figure 1:**
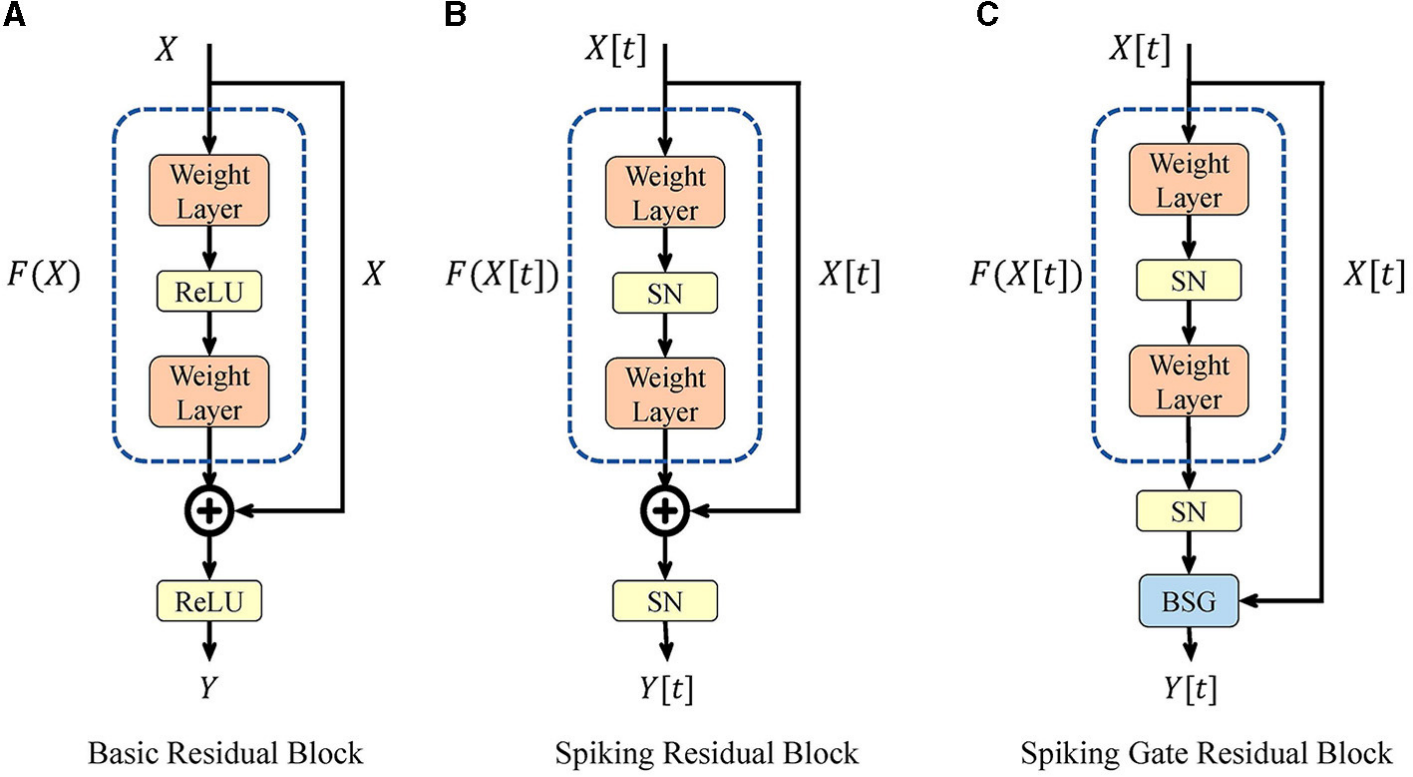
Basic residual block **(A)**, spiking residual block **(B)**, and our spiking gate residual block **(C)**.

The high-level architecture of SG ResNet is the same as that of ResNet. We used the spiking gate residual block to replace the base module and created a deep SNN without gradient vanishing. Figure 1 shows the structures of the basic residual block, spiking residual block, and spiking gate residual block.

#### 3.2.1. Basic and spiking residual block

Figure 1A shows the basic residual block in ResNet, where ReLU represents the rectified linear unit activation function, and the weight layer consists of a convolutional layer and a batch normalization layer. This expression is given by Eq. 10, where *X* is the input of the current block, which is the output of the previous block. Based on the property of ReLU function in the domain [0, +∞), the residual block can easily implement identity mapping when F(X)≡0.


(10) $$\begin{array}{l} Y=\mathrm{ReLU}\left(F\left(X\right)+X\right) \end{array}$$

Figure 1B shows the structure of the spiking residual block, which replaces ReLU function with spiking neurons. Its expression is given in Eq. 11. Fang et al. (2021a) proved that, in such a structure, identity mapping could only be achieved by using the IF model under specific conditions, leading to gradient vanishing or explosion in deep spiking ResNet.


(11) $$\begin{array}{l} Y\left[t\right]=\mathrm{SN}\left(F\left(X\left[t\right]\right)+X\left[t\right]\right) \end{array}$$

#### 3.2.2. Spiking gate residual block

**Figure 2:**
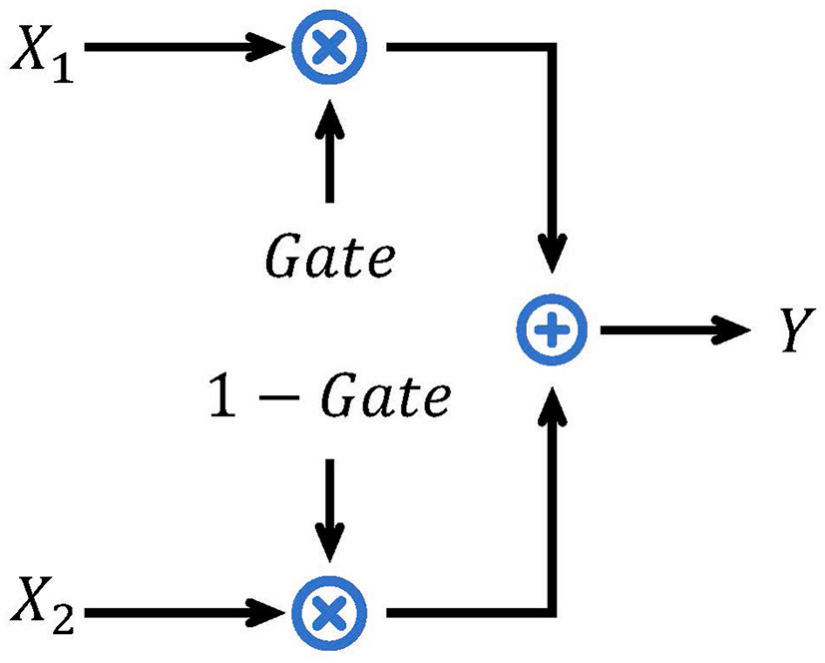
Illustration of the gate mechanism.

The basic gate mechanism is shown in Figure 2. Given inputs *X*_1_, *X*_2_, and the gate signal *Gate* ∈ [0, 1], the analog gate mechanism performs a weighted sum of *X*_1_ and *X*_2_. If a binary value is selected as *Gate* and both inputs are spike signals, the gate mechanism chooses one of the inputs as the output. We call it a binary selection gate (BSG). The formula of BSG is shown as follows:


(12) $$\begin{array}{l} Y=BSG(X_{1},X_{2};Gate)\\ \;=Gate\cdot X_{1}+(1-Gate)\cdot X_{2}. \end{array}$$

Figure 1C displays the structure of our spiking gate residual (SGR) block, where *X*[*t*] and *Y*[*t*] are the input and output of the module, respectively. SN is a spiking neuron layer. There are two main modifications compared to spiking residual blocks. First, the second SN is moved before the shortcut connection such that no redundant activation of *X*[*t*] is performed, similar to the ReLU before addition (RBA) block (He et al., 2016). Second, the addition operation of the shortcut connection is replaced by the BSG, and the output and intermediate variables are always spiking signals with the help of the binary signal *Gate*. The formulation of this block is expressed in Eq. 13. When *Gate* equals 1, the output is SN(F(X[t])), and when *Gate* is 0, the output is *X*[*t*], with the SGR block implementing identity mapping. The formulation of *Gate* will be discussed later.


(13) $$\begin{array}{l} Y[t]=\mathrm{BSG}\left(\mathrm{SN}\left(F\left(X[t]\right)\right)\text{​},X[t];Gate\right)\;\\ \;=Gate\cdot \mathrm{SN}\left(F\left(X[t]\right)\right)+(1-Gate)\cdot X[t]. \end{array}$$

In the basic residual block, at least one ReLU activation exists between the input and the output. Specific properties of the ReLU function make multiple activations equivalent to a single activation. Moreover, multiple activations have the benefit of preventing infinite output in deep layers, which exists in the RBA block. However, properties of spiking neurons differs from ReLU. Multiple SN activations are not equivalent to a single activation and may even block gradient propagation. Therefore, to remove the redundant activations, the second SN is moved to the position before the shortcut connection in Eq. 13. Meanwhile, our BSG ensures that the module's output remains spiking signal, which avoids the infinite output in the RBA block.

#### 3.2.3. Formulation of downsample block

**Figure 3:**
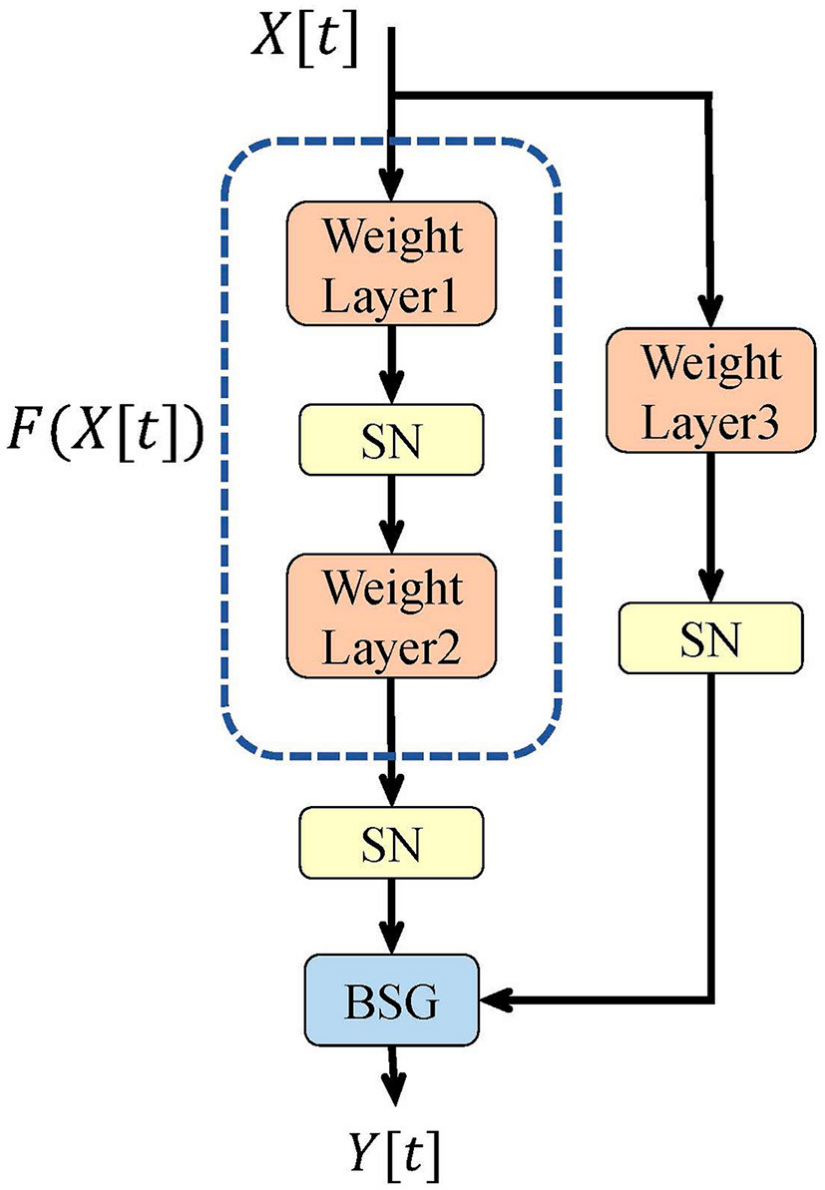
Downsample spiking gate residual block in SG ResNet.

As a stack of the above SGR blocks, the SG ResNet usually consists of multiple stages. In some stages, the first block must downsample the image, and hence, it is referred to as a downsample block, and its structure is shown in Figure 3. We replace the original identity connection with the third weight layer and an SN neuron layer in this block. Meanwhile, to realize downsampling, a convolutional layer with a stride of 2 is adopted in the first and third weight layers. Due to SN activation in the shortcut path, this downsample block cannot achieve perfect identity mapping. However, the number of downsample blocks is usually only four and stays unchanged as the depth of the network grows. Therefore, the corresponding degradation is ignored.

### 3.3. Gate formulation and analysis

#### 3.3.1. Formulation of binary selection gate

The most important part of the BSG module is *Gate*, the binary selection gate signal. For convenience, we denote the hidden features after the second weight layer of the SGR Block as *H*[*t*], where H[t]=SN(F(X[t])). *H* ∈ *R*^*T*×*c*×*h*×*w*^ stacks all features over the temporal dimension. In this study, *T* represents the time step of the spiking network, *c* is the number of channels of the feature map, and *h* and *w* are the height and width of the feature map, respectively. The expression for *Gate* is provided in Eq. 14:


(14) $$\begin{array}{l} Gate=\text{Θ}\left(W\cdot H+B-thr\right), \end{array}$$

where *W, B* ∈ *R*^*T*×*c*×*h*×*w*^. In Eq. 14, we first applied element-wise linear mapping to *H* with learnable weight *W* and bias *B*. Then, the heaviside step function with a threshold of *thr* shown in Eq. 4 is applied to transform the analog result into binary form. *W* and *B* are initialized to 1 and 0, respectively. Under such conditions, when *H* = 0, the value of *Gate* will also be zero, and the entire SGR block will act as an identity mapping block. In practice, *h* and *w* vary with the image size, whereas *T* changes with the simulation time step. If the image size or simulation time step is large, the number of parameters corresponding to *W* and *B* may be unacceptable. For efficiency, we proposed two representations: BSG* with learnable parameters and BSG without learnable parameters.

For BSG*, we have *H* share weight and bias along the dimensions *T*, *h*, and *w*. Therefore, the sizes of *W* and *B* are *R*^1 × *c*×1 × 1^.

For BSG, we have *Gate* = *H* when *W*≡1 and *B*≡0. Under such conditions, *W* and *B* are constant, and the SGR block has no additional learnable parameters. In this case, the SGR block can be expressed as follows:


(15) $$\begin{array}{l} Y=Gate\cdot H+(1-Gate)\cdot X\\ \;=H^{2}+(1-H)X. \end{array}$$

#### 3.3.2. Gradient analysis

With BSG and BSG*, the SGR block achieves identity mapping when *H*≡0. In this case, the gradient of the SGR block's output *Y* with respect to input *X* can be calculated using the following formula. Because the second SN has been moved before the shortcut connection, the following gradients do not need to involve the derivation of the spiking neurons.


(16) $$\begin{array}{l} \frac{\partial Y}{\partial X}=\frac{\partial (Gate\cdot H+(1-Gate)\cdot X)}{\partial X}\\ \;=\frac{\partial (0+1\cdot X)}{\partial X}=1. \end{array}$$

Denote *Y*^*l*^ and *X*^*l*^ as the output and input of the *l*_*th*_ block, respectively. As *Y*^*l*^ = *X*^*l*+1^, the gradient backpropagation can be calculated as follows:


(17) $$\begin{array}{l} \frac{\partial Y^{l+k}}{\partial X^{l}}=\overset{k}{\underset{i=0}{\prod }}\frac{\partial Y^{l+i}}{\partial X^{l+i}}=1. \end{array}$$

Because the relative gradient above is constant, the gradient of the deep layers in SG ResNet can be backpropagated to the shallow layers. Thus, SG ResNet can solve the gradient vanishing or explosion problem.

#### 3.3.3. Difference to SEW ResNet

SG ResNet and SEW ResNet (Fang et al., 2021a) are both improved variants of spiking ResNet, while their motivations are different, which is ultimately reflected in the different ways of integrating the left and right branches of the residual block.

SEW ResNet aims to achieve identity mapping, using element-wise functions to integrate the left and right branches. It proposes spike-constrained AND and IAND functions and the unconstrained ADD function. However, the ADD function requires non-spike computations, making it unsuitable for deployment.

We plan to use the gate mechanism to control the flow of spike information. At the beginning of the design, we set both spike constraint and identity mapping as goals. First, we binarize the analog gate signal into a spike one, and the gate mechanism turns into a binary selection gate, which can ensure that the output is also a spike signal. Furthermore, we have proven that when the selection of the gate signal comes from the hidden feature H of the left branch, and the residual block can achieve identity mapping.

From the performance perspective, SG ResNet is superior to SEW ResNet based on AND and IAND functions under spike constraint. However, if the spike condition is relaxed, SEW ResNet based on the ADD function is better. The ADD function integrates both branches without loss of information, while the spike constraint determines that information integration is inevitably lossy. This comparison will also be analyzed in the experiment.

### 3.4. Attention spike decoder

**Figure 4:**
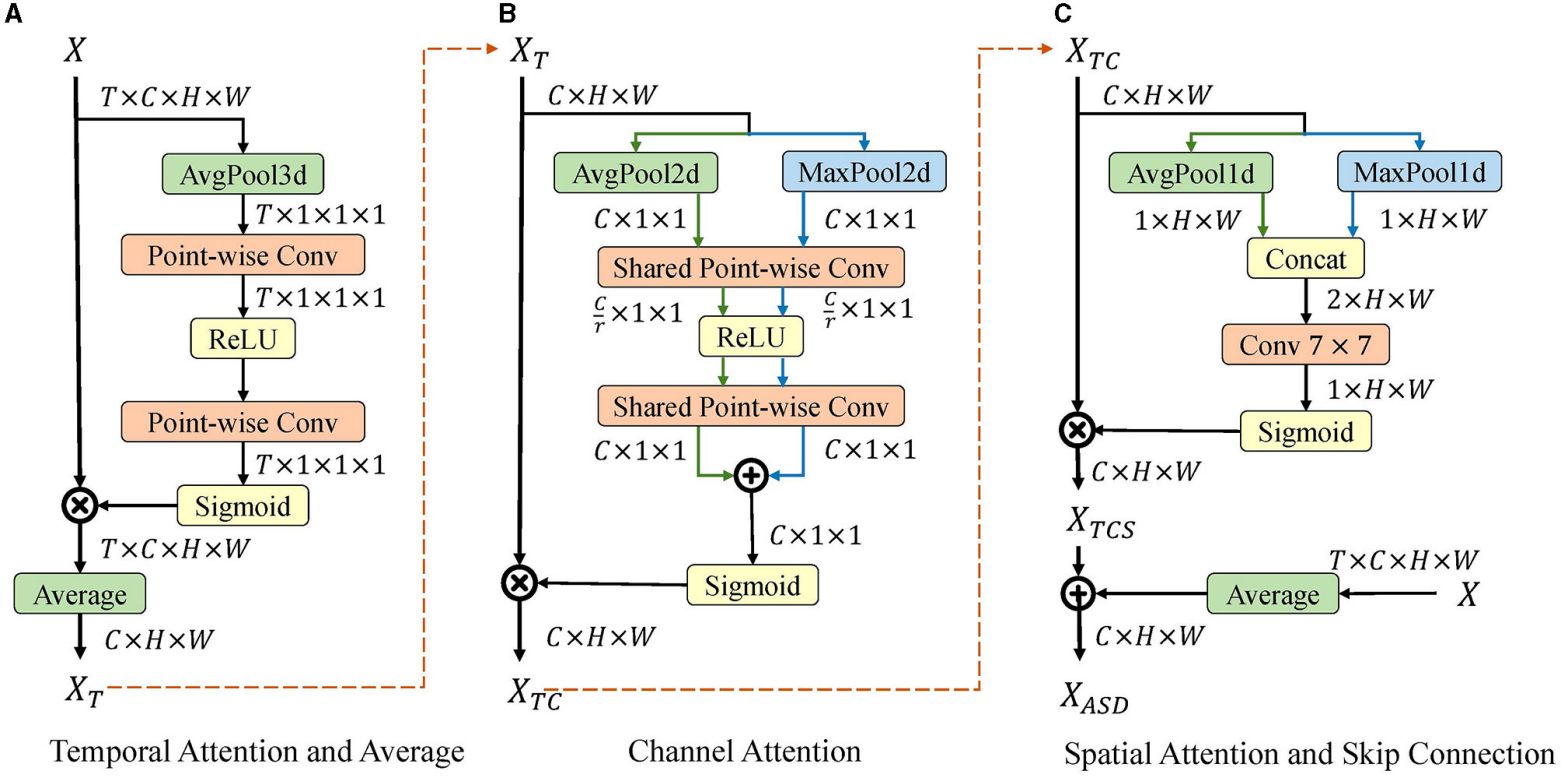
Illustration of attention spike decoder. Given spiking feature *X*, temporal-wise refinement is first performed using temporal attention, and the features along time dimension are averaged in **(A)**. Then, channel attention **(B)** and spatial attention **(C)** perform feature refinement sequentially. Finally, the output of rate coding and spatial attention is added for the output.

After the spiking network, a decoder is required to decode the spiking features to analog. The spiking feature output by the SG ResNet is denoted as *X* ∈ *R*^*T*×*c*×*h*×*w*^, where *T* is the time steps, *c* is the channel size, and *h* and *w* denote the spatial dimension of the image. The rate coding method averages *X* along the temporal dimension, so the resulting $X_{R}\in R^{c\times h\times w}$ is the corresponding firing rate. However, such a method treats information at each time point as equally important, which is not reasonable in neuroscience. We introduce the attention module in Woo et al. (2018) along multiple dimensions and propose ASD module to decode the spiking features and fully utilize the information in the sparse spike form. The detailed structure of the ASD module is shown in Figure 4, including temporal, channel, and spatial attention, as well as the averaging operation and skip connections. Given spiking feature *X*, we first perform temporal-wise refinement using temporal attention (TA) and then average the feature along the time dimension. Channel attention (CA) and spatial attention (SA) then perform feature refinement sequentially. Finally, the output of the rate coding and SA are added as the output of ASD by skip connections.

#### 3.4.1. Temporal attention and average operation

As is shown in Figure 4A, TA first computes channel-spatial statistics using 3-D global average-pooling. These statistics are fed into two point-wise convolutional layers to obtain the 1-D temporal attention map. Subsequently, after a sigmoid function, the 1-D temporal attention map is multiplied by the origin feature.

Before the output of TA is sent to CA, the average operation is used to squeeze features in the temporal dimension for two reasons. First, a squeeze of the temporal dimension by the average operation significantly reduces the computational effort of TA and SA while keeping the statistics and attention maps unchanged. Second, both TA and SA use max-pooling to extract attention maps. Each element of the spiking feature is a Boolean value that is unsuitable for max-pooling. Therefore, Boolean values are converted to analog before TA and SA.

#### 3.4.2. Channel attention

As is shown in Figure 4B, CA takes *X*_*T*_ as the input and outputs channel-refined feature *X*_*TC*_. First, we extract spatial statistics using 2-D global average-pooling and max-pooling. These two sets of statistics are then fed into a two-layer point-wise convolution with shared weights. The output channel size for the first convolution is set to *C*/*r* to reduce computation, where *r* is the reduction rate. After the convolution, the two outputs are merged by element-wise summation and a channel attention map is generated using sigmoid activation. Finally, the channel-refined feature *X*_*TC*_ is obtained by multiplying the attention map with *X*_*T*_.

#### 3.4.3. Spatial attention and skip connection

As is shown in Figure 4C, SA takes *X*_*TC*_ as the input and outputs the spatial-refined feature *X*_*TCS*_. First, we extract and concatenate the channel statistics using 1-D average-pooling and max-pooling. The concatenated statistics are then fed into a 7 × 7 convolutional layer with a sigmoid activation to generate a spatial attention map. Finally, the spatially refined feature *X*_*TCS*_ is obtained by multiplying the attention map with *X*_*TC*_.

Instead of directly using *X*_*TCS*_ as the output, we add it to the rate coding feature by the skip connection to obtain a more robust feature representation. The average operation is utilized to obtain the rate coding feature from the original spiking feature. Finally, the output *X*_*ASD*_ of the ASD module is the fusion of attention-refined and rate-coded features.

## 4. Object recognition and detection

**Figure 5:**
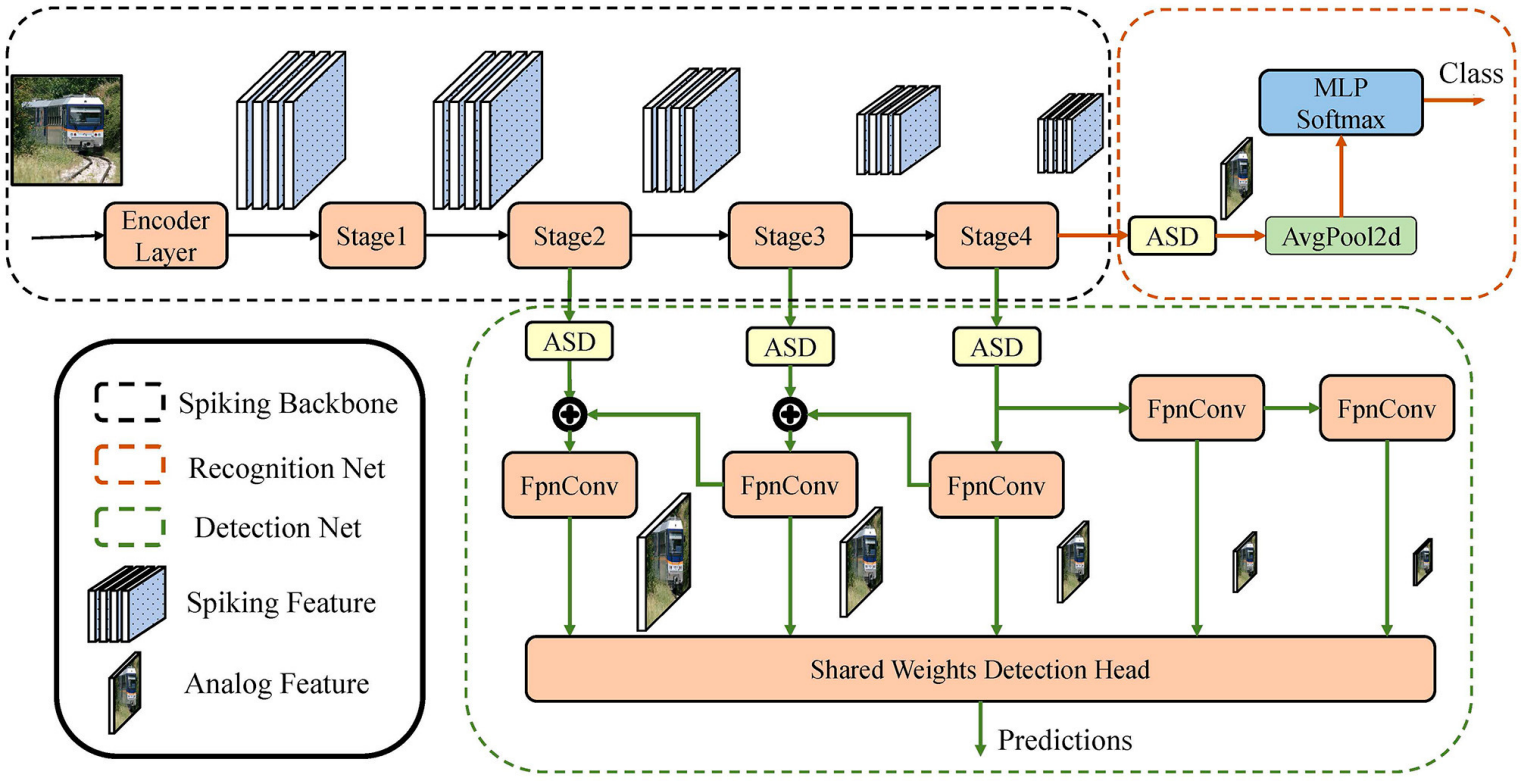
Illustration of the networks in object recognition and object detection tasks. The object recognition network consists of the spiking backbone and recognition network; the object detection network consists of the spiking backbone and detection network.

Object recognition requires the network to output the semantic class of the specified image, and object detection requires bounding boxes and corresponding classes of all objects in the image. In this section, we explain our network structure that solve these two problems, as is shown in Figure 5. The network is composed of the spiking backbone, recognition network, and detection network. The proposed SG ResNet is used as a spiking backbone to extract the image features. The recognition network decodes the image features and obtains the corresponding semantic classes. The detection network takes multiple scales of image features as inputs and outputs the bounding boxes and corresponding classes of objects. Thus, the object recognition network consists of a spiking backbone and a recognition network; the object detection network consists of the spiking backbone and a detection network.

### 4.1. Spiking backbone

The spiking backbone consisted of an encoder layer and a four-stage computational body. The encoder layer includes a convolution with a stride of 2, an IF neuron, and a max-pooling downsampling layer. It receives analog images as inputs and converts them into spikes. The four-stage body is built with spiking gate residual blocks and performs the bulk of computation. The second to fourth stages downsample the features and output 8, 16, and 32 times downsampled spiking features, respectively. The detailed kernel, depth, and channel size settings of the spiking backbone are listed in Table 1. The layer configurations refers to the classic ResNet configurations. As a result, the amount of layers includes the total number of convolutional layers in the backbone and the fully connected layer in the recognition network.

**Table 1:** Detailed network settings of SG ResNet.

	**10-layers**	**18-layers**	**34-layers**	**50-layers**
Encoder layer	7 × 7, 64, stride 2 for ImageNet and MSCOCO/3 × 3, 64, stride 1 for CIFAR			
		3 × 3 maxpool, stride 2 for ImageNet and MSCOCO/identity for CIFAR			
Stage1	$\left(\begin{array}{c} 3\times 3,643\times 3,64 \end{array}\right)\times 1$	$\left(\begin{array}{c} 3\times 3,643\times 3,64 \end{array}\right)\times 2$	$\left(\begin{array}{c} 3\times 3,643\times 3,64 \end{array}\right)\times 3$	$\left(\begin{array}{c} 1\times 1,643\times 3,641\times 1,256 \end{array}\right)\times 3$
Stage2	${\left(\begin{array}{c} 3\times 3,1283\times 3,128 \end{array}\right)}^{*}\times 1$	${\left(\begin{array}{c} 3\times 3,1283\times 3,128 \end{array}\right)}^{*}\times 2$	${\left(\begin{array}{c} 3\times 3,1283\times 3,128 \end{array}\right)}^{*}\times 4$	${\left(\begin{array}{c} 1\times 1,1283\times 3,1281\times 1,512 \end{array}\right)}^{*}\times 4$
Stage3	${\left(\begin{array}{c} 3\times 3,2563\times 3,256 \end{array}\right)}^{*}\times 1$	${\left(\begin{array}{c} 3\times 3,2563\times 3,256 \end{array}\right)}^{*}\times 2$	${\left(\begin{array}{c} 3\times 3,2563\times 3,256 \end{array}\right)}^{*}\times 6$	${\left(\begin{array}{c} 1\times 1,2563\times 3,2561\times 1,1,024 \end{array}\right)}^{*}\times 6$
Stage4	${\left(\begin{array}{c} 3\times 3,5123\times 3,512 \end{array}\right)}^{*}\times 1$	${\left(\begin{array}{c} 3\times 3,5123\times 3,512 \end{array}\right)}^{*}\times 2$	${\left(\begin{array}{c} 3\times 3,5123\times 3,512 \end{array}\right)}^{*}\times 3$	${\left(\begin{array}{c} 1\times 1,5123\times 3,5121\times 1,2,048 \end{array}\right)}^{*}\times 3$

^*^Represents that the first SGR block performs downsampling.

### 4.2. Recognition network

The recognition network has a small number of parameters. First, the ASD module is used to decode the output of the fourth stage in the backbone into the corresponding analog feature. Then, the analog image feature is squeezed into a 1-D vector by global average-pooling. A fully connected layer followed by a softmax function classifies the vector into the semantic class, which performs recognition of the images.

### 4.3. Detection network

The proposed spiking RetinaNet, a hybrid SNN-ANN object detector, consists of an SG ResNet (SNN) and a detection network (ANN). SG ResNet extracts the deep features of images for detection network, and the ASD module does the intermediate signal conversion. RetinaNet is chosen as our detection framework because it is one of the most classic one-stage detectors. Its backbone and detection subnet are highly decoupled. It fits well with most ResNet-like backbones, making it very suitable for verifying and comparing the feature extraction capabilities of the backbones in detection tasks.

In this study, the feature pyramid petwork (FPN) (Lin et al., 2017a) and detection head in the RetinaNet (Lin et al., 2017b) model are used for the detection network. First, we use three independent ASD modules to convert the spiking features of the second to fourth stages in the backbone into analog features. Taking these features as the input, the FPN constructs a five-level feature pyramid with levels from *P*_3_ to *P*_7_, where the resolution of *P*_*l*_ is 2^*l*^ times lower than the input, and each *P*_*l*_ has 256 channels. The feature pyramid is fed into a detection head with shared weights between different scales. The detection head consists of two sub-networks, the classification subnet and box regression subnet, each having four convolutional layers to accomplish the corresponding task. After the processing of the two sub-networks, the final predictions are obtained, including the bounding box and class information of the object.

## 5. Experiment

The methods are tested on the object recognition and detection tasks. For object recognition, SG ResNet is compared with other methods on both the static and neuromorphic image benchmarks, including CIFAR-100, CIFAR-10, ImageNet, and DVS-CIFAR10. For object detection, we demonstrate that the performance of our spiking RetinaNet is very close to that of RetinaNet with artificial neurons under the same experimental setup. Ablation studies are then conducted on several vital questions, such as the ability to overcome gradient vanishing.

Since both SG ResNet and SEW ResNet are variants of Spiking ResNet and SEW ResNet based on ADD does not strictly meet the spike constraints, we compare the two methods in the ablation study and show the advantages of SG ResNet under the condition of spike constraints.

In our experiments, the implementation and GPU acceleration of all neurons are based on the PyTorch and SpikingJelly (Fang et al., 2020) frameworks. On natural image datasets, we adopt IF as the spiking neuron and set *V*_*rest*_ = 0 and *V*_*th*_ = 1. For the DVS-CIFAR10 dataset, we adopt the PLIF neuron and set the initial time constant to 2. The ArcTan function ($\sigma^{\prime }\left(x\right)=\frac{1}{1+{\left(\pi x\right)}^{2}}$) is used as the surrogate function to calculate the gradients of all spiking neurons. For all experiments, we use the stochastic gradient descent (SGD) optimizer with a momentum of 0.9. To reduce GPU memory cost and accelerate training, we adopt the mixed precision training in PyTorch. The training schedule, learning rate, batch size, and other parameters are presented in Table 2.

**Table 2:** Training settings and hyper parameters.

**Dataset**	**Learning rate schedule**	**Epoch**	**Learning rate**	**Weight decay**	**Batch size**	**GPU**
CIFAR-100	Step, *T*_*steps*_ = [60, 120, 160]	200	0.1	0.0001	32	1
CIFAR-10	Step, *T*_*step*_ = [100, 150]	200	0.1	0.0001	32	1
ImageNet	Cosine, *T*_*max*_ = 320	320	0.1	0	32	8
DVS-CIFAR10	Cosine, *T*_*max*_ = 64	64	0.01	0	8	1
MSCOCO	Step, *T*_*step*_ = [64, 70]	72	0.01	0.0001	2	8

### 5.1. Object recognition

Comparisons on CIAFR-100, CIFAR-10, ImageNet, and DVS-CIFAR10 are presented in Table 3. Unless otherwise specified, the decoding modules used after SG ResNet are ASD modules. We list the deploying methods of all studies. Among them, the spike-based BP means the direct-training method using the surrogate gradient. ANN-to-SNN means the ANN-to-SNN conversion method. Hybrid training combines the above two methods and trains networks in two stages, and IDE training, tandem learning, and SNN distillation are specialized training methods designed for SNNs.

**Table 3:** Top-1 accuracy and time step comparisons on object recognition datasets.

**Dataset**	**Network**	**Deploying methods**	**Time steps**	**Accuracy**
CIFAR-100	ResNet20 (Han et al., 2020)	ANN-to-SNN	4,096	67.82%
		VGG-like (Yan et al., 2021)	ANN-to-SNN	300	71.84%
		ResNet20 (Liu et al., 2022)	ANN-to-SNN	16	68.69%
		VGG11 (Rathi et al., 2020)	Hybrid Training	125	67.84%
		CIFARNet-F (Xiao et al., 2021)	IDE Training	100	73.07%
		Ms ResNet110 (Hu et al., 2021)	Spike-based BP	–	66.83%
		AutoSNN (Na et al., 2022)	Spike-based BP	8	69.16%
		SNASNet (Kim et al., 2022)	Spike-based BP	8	73.04%
		MT-ResNet-20 (Wang et al., 2023)	Spike-based BP	5	73.45%
	SG ResNet10 (ours)	Spike-based BP	4	73.19%
	SG ResNet18 (ours)	Spike-based BP	4	74.86%
	SG ResNet34 (ours)	Spike-based BP	4	75.64%
CIFAR-10	ResNet20 (Han et al., 2020)	ANN-to-SNN	4,096	91.36%
		VGG16 (Rathi et al., 2020)	Hybrid training	200	92.02%
		CIFARNet (Wu et al., 2021)	Tandem learning	8	90.98%
		ResNet18 (Xu et al., 2023b)	SNN Distillation	4	93.41%
		ResNet19 with tdBN (Zheng et al., 2021)	Spike-based BP	6	93.16%
		Ms ResNet110 (Hu et al., 2021)	Spike-based BP	–	92.12%
		AutoSNN (Na et al., 2022)	Spike-based BP	8	93.15%
	SNASNet (Kim et al., 2022)	Spike-based BP	8	94.12%
	DS-ResNet (Feng et al., 2022)	Spike-based BP	4	94.25%
	MT-ResNet-20 (Wang et al., 2023)	Spike-based BP	5	94.44%
	SG ResNet10 (ours)	Spike-based BP	4	93.0%
	SG ResNet18 (ours)	Spike-based BP	4	93.92%
	SG ResNet34 (ours)	Spike-based BP	4	94.52%
ImageNet	ResNet34 (Han et al., 2020)	ANN-to-SNN	4,096	69.89%
		ResNet20 (Li et al., 2021)	ANN-to-SNN	32	64.54%
		ResNet34 (Rathi et al., 2020)	Hybrid Training	250	61.48%
		ResNet34 with tdBN (Zheng et al., 2021)	Spike-based BP	6	63.72%
		ResNet50 with tdBN (Zheng et al., 2021)	Spike-based BP	6	64.88%
		Ms ResNet34 (Hu et al., 2021)	Spike-based BP	6	69.42%
		SG ResNet18 (ours)	Spike-based BP	4	62.51%
		SG ResNet34 (ours)	Spike-based BP	4	65.08%
		SG ResNet50 (ours)	Spike-based BP	4	66.25%
	DVS-CIFAR10	CIFARNet (Wu et al., 2021)	Tandem learning	20	65.59%
		7-layer CNN (Wu et al., 2019)	Spike-based BP	40	60.50%
		ResNet-19 (Zheng et al., 2021)	Spike-based BP	10	67.80%
		Ms ResNet20 (Hu et al., 2021)	Spike-based BP	–	75.56%
	AutoSNN (Na et al., 2022)	Spike-based BP	20	72.50%
	DS-ResNet (Feng et al., 2022)	Spike-based BP	–	70.36%
	7B SG ResNet (ours)	Spike-based BP	16	70.60%

For a fair comparison, we use the standard top-1 accuracy in the object recognition task for all datasets. Top-k accuracy is an essential metric for assessing model generalization ability and refers to the proportion of samples in the test set for which the correct category appears in the top-k confidence of the model's output. The higher the metric, the better the model performs.

#### 5.1.1. CIFAR-100

CIFAR-100 is a static image classification dataset with 60,000 images and a image size of 32 × 32. It contains 100 classes, and each class has 500 images for training and 100 images for testing. On the CIFAR-100 dataset, we apply random cropping with a size of 32, a padding with a size of 4, and horizontal flipping for data augmentation. Moreover, data normalization is applied by subtracting the mean value of the pixel intensity and dividing by the standard variance. This ensures that the input images have zero mean and unitary variance.

We make some modifications to the SG ResNet for the CIFAR dataset by setting the kernel size of the first convolutional layer to 3 × 3 and removing the max-pooling layer at the same time. We test it on three networks with different depths, SG ResNet10, SG ResNet18, and SG ResNet34. As is expected, the greater the network depth, the higher the accuracy. Thus, SG ResNet does not suffer from the gradient vanishing problem. We achieve 75.64% accuracy with a time step of only 4 on the SG ResNet34 network, which is much better than the other methods in terms of both latency and performance.

#### 5.1.2. CIFAR-10

CIFAR-10 is a small-size dataset similar to CIFAR-100. It has only 10 classes, and each class contains 5,000 images for training and 1,000 images for testing. Data augmentation and pre-processing on CIFAR-10 are the same as CIFAR-100 dataset.

On CIFAR-10, we adopted a network structure similar to CIFAR-100 and conducted experiments on the three depths. Compared with other network-structure-level methods of SNNs, we achieve 94.52% accuracy with a time step of 4 on the SG ResNet34 network. This confirms the superiority of our newly proposed method.

#### 5.1.3. ImageNet

ImageNet (Deng et al., 2009) is a large-scale dataset which contains 1.28 million images for training and 50,000 images for validation. On this dataset, we randomly crop the images with a size of 224 × 224. Furthermore, random horizontal flipping is further applied for augmentation. Similar to CIFAR-100 and CIFAR-10, we normalize every image to ensure zero mean and unitary variance.

On ImageNet, we conduct experiments on SG ResNet18, SG ResNet34, and SG ResNet50. With the ASD module, SG ResNet34 has achieved an accuracy of 65.08%. Furthermore, SG ResNet50 has achieved 66.25% accuracy with a time step of only 4. Here, SG ResNet50 uses rate coding as the decoder because we found that SG ResNet50 with ASD decoder converges much faster than rate coding, indicating an overfitting problem in the training process. Same as that in CIFAR datasets, we observe an enhancement of accuracy in deeper networks. Our SG ResNet outperforms some studies with spike-based BP and hybrid training methods. Compared with ANN-to-SNN methods, we report competitive results with much fewer time steps.

#### 5.1.4. DVS-CIFAR10

DVS-CIFAR10 (Li et al., 2017) is a neuromorphic dataset which contains 10,000 images in the format of spike train. It is obtained by recording the moving images of CIFAR-10 on a LCD monitor with a DVS camera. On this dataset, we adopt the AER data pre-processing (Fang et al., 2021b). During the pre-processing, the event is split into 16 slices(same number as time steps). Furthermore, for each training sample, we randomly deleted four slices for augmentation. We use a 7B-Net which contains seven SGR blocks, which has achieved 70.6% accuracy with a time step of 16.

### 5.2. Object detection

We validate the effectiveness of spiking RetinaNet on the MSCOCO (Lin et al., 2014) dataset and compare it with ANN RetinaNet. MSCOCO is a large-scale object detection dataset containing 330K images and 80 target classes. In the experiments, the train and val sets of the 2017 release are used as our training and testing datasets, respectively. We randomly crop the images with a size of 1,333 × 800. Furthermore, random horizontal flipping with a ratio of 0.5 was applied for augmentation during training.

Two metrics, mean average precision (*mAP*) and mean average recall (mAR), are used to evaluate object detection effectiveness. *AP* evaluates the ability of the detector to perform correct classification and accurate localization of a certain category. *mAP* is the average value of *AP* for each category. In addition to the *mAP*, we evaluate the detection performance for objects of different sizes. *AP*_*S*_, *AP*_*M*_, and *AP*_*L*_ indicate the detection performance for small, medium, and large objects, respectively. Unlike precision, recall is concerned with whether the detector can detect more ground truths. *AR* is the average recall over IoU from 0.5 to 1.0. Similarly, we also use *AR*_*S*_, *AR*_*M*_, and *AR*_*L*_ to represent the recall of small, medium, and large objects, respectively.

The training schedules are listed in Table 2. Time steps of all SG ResNet backbones are 4. The experimental results are presented in Table 4. For each comparison group, the largest *mAP* and *mAR* are in bold, and the second largest *mAP* and *mAR* are underlined.

**Table 4:** Object detection results of ANN RetinaNet and spiking RetinaNet on the MSCOCO dataset.

**Network**	**Backbone**	**With *ASD***	***mAP***	***AP*_*S*_**	***AP*_*M*_**	***AP*_*L*_**	***mAR***	***AR*_*S*_**	***AR*_*M*_**	***AR*_*L*_**
ANN RetinaNet	ANN ResNet18	✗	**0.299**	0.143	0.313	0.417	**0.478**	0.269	0.502	0.659
Spiking RetinaNet	SG ResNet18	✗	0.280	0.141	0.290	0.395	0.468	0.259	0.497	0.636
Spiking RetinaNet	SG ResNet18	✓	0.285	0.150	0.300	0.400	0.476	0.272	0.508	0.643
ANN RetinaNet	ANN ResNet34	✗	**0.319**	0.159	0.339	0.448	**0.497**	0.289	0.523	0.680
Spiking RetinaNet	SG ResNet34	✗	0.292	0.148	0.308	0.406	0.478	0.268	0.510	0.653
Spiking RetinaNet	SG ResNet34	✓	0.296	0.153	0.313	0.407	0.484	0.280	0.510	0.657

It is worth mentioning that SG ResNet consumes tens of times less energy than ANN ResNet, which is quantitatively analyzed in Section 5.3.1. With such energy efficiency, spiking RetinaNet can accomplish the object detection task effectively. Under the condition of using backbones of the same depth, spiking RetinaNet achieved a slightly lower *mAP* than ANN RetinaNet but with a far more energy-efficient backbone. Spiking RetinaNet with SG ResNet18 and ASD module achieved an mAP of 0.285 and mAR of 0.476. By comparing the detection results of different objects, we find that spiking RetinaNet is more robust in detecting small objects. Compared with the median and large objects, the performance degradation of small objects is lower. The *AP*_*S*_ and *AR*_*S*_ of spiking RetinaNet with SG ResNet18 and ASD are even higher than those of ANN RetinaNet.

### 5.3. Ablation studies

#### 5.3.1. Energy efficiency comparison

The energy efficiency of SG ResNet is analyzed in the study. The network energy consumption is related to the type of operations it employs and the number of floating-point operations (FLOPS). Most operations in the convolutional layers of ANNs are multiply-and-accumulate (MAC) (Panda et al., 2020). However, because the spiking signals used by SNNs are binary, the convolutional layers of SNNs use only the accumulate (AC) operations. These operations occur only when the spiking neuron fires a spike. Certainly, some layers will also adopt MAC operations in SNNs, such as the encoder layer and the ASD module in SG ResNet. The FLOPS counts of the convolutional layers of ANN and SNN for CIFAR-100 are calculated using Eqs 18 and 19, respectively.


(18) $$\begin{array}{l} FLOPS_{ANN}=O^{2}\times C_{in}\times C_{out}\times k^{2}, \end{array}$$

(19) $$\begin{array}{l} FLOPS_{SNN}=O^{2}\times C_{in}\times C_{out}\times k^{2}\times Fr\times T, \end{array}$$

where *O* is the output size, *C*_*IN*_ and *C*_*out*_ denote the input and output channel size, and *k* is the weight kernel size. Because the spike activity of SNN is sparse, the firing rate *Fr*≪1 in each convolutional layer. For the energy calculation, we take the energy consumption of 45nm COMS technology (Han et al., 2015) as the criterion, in which 32-bit integer MAC operation consumes 3.1pJ, and 32-bit integer AC operation consumes 0.1pJ. We calculate the FLOPS of MACs and ACs in SNN and ANN, respectively, and further estimate the total energy consumption. Using 3 × 32 × 32 CIFAR images as the input, we analyzed ANN ResNet18 and SG ResNet18. Because the number of parameters and FLOPs of batch normalization are small and can be incorporated into the convolutional layer during deployment, we ignored the effect of batch normalization in our experiments. Analysis results are shown in Table 5.

**Table 5:** Energy efficiency comparison between ANN ResNet18 and SG ResNet18.

**Network**	**Parameters**	**MACs**	**ACs**	**Energy**
ANN ResNet18	11.22M	549.2M	0M	1.7 × 10^9^pJ
SG ResNet18	11.25M	1.9M	348.9M	4.1 × 10^7^pJ

Regarding parameter numbers, SG ResNet18 has only 0.03M more than ANN ResNet18 (from the ASD module). In terms of FLOPS, SG ResNet18 has only 1.9M MACs, and most of the operations are ACs. The total energy consumed by SG ResNet18 is 4.1 × 10^7^pJ, which is 41.7 times lower than that of ANN ResNet18.

#### 5.3.2. Effects of the ASD module

We propose the attention spike decoder to convert image features from spiking to analog. In this part, we validate the superiority of the ASD module over the rate coding method in SG ResNet10, SG ResNet 18, and SG ResNet 34. Experiments are conducted on CIFAR-100, and the training setups of the two decoders are the same. Results are presented in Table 6.

**Table 6:** Top-1 accuracy comparisons between rate coding method and our attention spike decoder on CIFAR-100 dataset.

**Network**	**Rate coding**	**Attention spike decoder**
SG ResNet10	72.68%	**73.19%**
SG ResNet18	74.62%	**74.86%**
SG ResNet34	75.01%	**75.64%**

The bold values indicate the maximum accuracies of the comparison.

In this experiment, the ASD module has only 0.033M parameters and 0.08M FLOPS, with an energy consumption of only 2.55 × 10^5^pJ (0.63% of the total energy consumption of SG ResNet18). This shows that the improvement brought by ASD is due to its superior design rather than the increase in parameters or computational power.

As is shown in the table, the attention spike decoder has improved the accuracy compared with rate coding. SG ResNet34 has the highest accuracy improvement among the three deep network structures, from 75.01 to 75.64%. The previous object detection experiments in Table 4 also show that the ASD module performs better than rate coding. In spiking RetinaNet with a SG ResNet18 backbone, the ASD module has improved the mAP and mAR by 0.5 and 0.8%, respectively. In the experiment of SG ResNet50 with ASD on ImageNet, an overfitting problem occurs, indicating that the ASD module may not fit well with large models with bottleneck modules.

#### 5.3.3. Validation on solving the gradient vanishing problem

The proposed SG ResNet solves the problem of gradient vanishing in spiking ResNet. Therefore, in this section, we compare SG ResNet with spiking ResNet in the structures of ResNet10, ResNet18, and ResNet34. The training setups of the two methods are the same. The experimental results are shown in Table 7. To eliminate the impact of the ASD module, rate coding is used for the decoding scheme in both networks.

**Table 7:** Top-1 accuracy comparisons between SG ResNet and spiking ResNet on the CIFAR-100 dataset.

**Network**	**SG ResNet**	**Spiking ResNet**
ResNet10	72.68%	**73.00%**
ResNet18	**74.62%**	74.36%
ResNet34	**75.01%**	32.05%

The bold values indicate the maximum accuracies of the comparison.

As is illustrated in Table 7, spiking ResNet has an acceptable accuracy over two relatively shallow network structures, ResNet10 and ResNet18. However, when the depth reached 34, gradient vanishing occurs. As the network depth increased from 18 to 34, the accuracy of spiking ResNet decreases from 74.36 to 32.05%. In contrast, with increase in depth, an enhancement is observed in accuracy of SG ResNet. Furthermore, our SG ResNet has the highest accuracy of 75.01% on the deepest ResNet34, thus proving that SG ResNet effectively solves the gradient vanishing problem.

#### 5.3.4. Comparison and discussion on SEW ResNet

Previously, SEW ResNet (Fang et al., 2021a) is also a variant of spiking ResNet that analyzed and solved the gradient vanishing problem from the perspective of residual learning. They analyzed the reason for the gradient vanishing theoretically and proposed the element-wise function to solve this problem. However, the most effective one of their proposed element-wise functions is ADD, which makes the network no longer spiking. In our SG ResNet, a gate mechanism is introduced to solve gradient vanishing while ensuring that the network is still spiking. In this section, we compare SG ResNet with SEW ResNet using IAND and ADD. Experimental results are shown in Table 8. To avoid the impact of the ASD module, the decoding scheme used in all methods is rate coding.

**Table 8:** Ablation study on the relationship between SG ResNet and SEW ResNet on CIFAR-100 dataset.

**Network**	**SG ResNet**	**SEW ResNet (IAND)**	**SEW ResNet (ADD)**
ResNet10	72.68%	71.96%	**73.02%**
ResNet18	74.62%	73.89%	**74.90%**
ResNet34	75.01%	73.8%	**75.93%**

Values in the table represents the top-1 accuracy. The bold values indicate the maximum accuracies of the comparison.

IAND is a binary operator that returns the inverse and of two inputs. The output of IAND operation with two spiking inputs remains a spiking signal. Thus, the SEW ResNet with IAND is a deployable network. Compared with SEW ResNet with IAND, our SG ResNet performs better at every depth. On the CIFAR-100 dataset, SG ResNet34 has achieved 1.21% higher accuracy than SEW ResNet34 (IAND). ADD is a binary operator that returns the addition of two inputs. However, SEW ResNet with ADD is more like an ANN rather than an SNN. As is expected, SEW ResNet (ADD) has the highest accuracy among the three methods.

Based on the above results, the SEW ResNet (ADD) structure, which is similar to the original ResNet, can achieve the best performance without considering signal type. However, this does not necessarily mean that it is the best. Our SG ResNet can be considered as an better compromise that improves accuracy while adhering to the spiking signal constraint.

#### 5.3.5. Comparison between BSG and BSG*

As is mentioned in Section 3.3, Eq. 14 is the general expression for our gate signal, and the module constituted by such *Gate* is BSG*. Furthermore, if the linear transformation in Eq. 14 is ignored and *Gate* directly equals *H*, then the module is of type BSG. This part compares these two modules, and the experimental results are shown in Table 9. To avoid the impact of the ASD module, rate coding is used as the decoding scheme for both methods. As is seen, both BSG and BSG* solve the gradient-vanishing problem. A deeper network brings the enhancement of accuracy. Through a lateral comparison, the network using BSG is more accurate than BSG*, primarily, for two reasons accounting. First, to reduce the number of parameters, *W* and *B* are only learnable in the channel dimension, which may lead to inaccuracy in the linear transformation. Second, we use heaviside step function to binarize the gate signal. During backpropagation, we use gradient surrogate functions, which may lead to inaccurate optimization of the gate signal. In summary, the effect of BSG* with linear transformation was not as good as that of BSG at present. We also hope that our research can help realize the effectiveness of the gate mechanism and further promote the detailed design.

**Table 9:** Ablation study on the binary selection gate formulations on CIFAR-100.

**Network**	**BSG**	BSG*
SG ResNet10	**72.68%**	71.75%
SG ResNet18	**74.62%**	73.71%
SG ResNet34	**75.01%**	74.21%

Values in the table represents the top-1 accuracy. The bold values indicate the maximum accuracies of the comparison.

## 6. Discussion and conclusion

This study focuses on the issues to be solved during direct training of high-performance SNNs in object recognition and detection tasks. We introduced a binary gate mechanism and presented the spiking gate ResNet to form deep architectures in SNNs. This is the first time that a widely used gate mechanism in RNNs is being combined with SNNs in the structural design. Through gradient analysis, we prove that SG ResNet can overcome gradient vanishing or explosion problems. An attention spike decoder is also proposed to address the spiking signal decoding problem. Using SG ResNet as the backbone and the ASD module for information decoding, we propose spiking RetinaNet, which is the first direct-training hybrid SNN-ANN detector for RGB images. The experimental results show that SG ResNet with an ASD decoder outperforms most direct-training SNNs with the surrogate gradient method on the object recognition task. Furthermore, spiking RetinaNet has achieved a satisfactory performance in object detection with an energy efficient spiking backbone.

Regarding the future research topics, the binary gate mechanism is non-trivial and valuable to be further explored, including the efficiency-performance trade-off of parameterized gate mechanism and binarization of gate signals. In addition, it will be quite helpful and contributive to investigate how to use gate mechanism in the residual connection of spiking transformer. Finally, downstream vision applications of spiking neural networks are also what we consider to be a crucial direction, including image segmentation, object detection, video recognition, optic flow estimation, and so on.

## Data availability statement

The original contributions presented in the study are included in the article/supplementary material, further inquiries can be directed to the corresponding author.

## Author contributions

HZ: methodology, software, and writing—original draft. YL: formal analysis and writing—review and editing. BH, XF, and YW: writing—review and editing. YZ: methodology and writing—review and editing. All authors contributed to the article and approved the submitted version.
